# Regularized machine learning on molecular graph model explains systematic error in DFT enthalpies

**DOI:** 10.1038/s41598-021-93854-w

**Published:** 2021-07-13

**Authors:** Himaghna Bhattacharjee, Nikolaos Anesiadis, Dionisios G. Vlachos

**Affiliations:** 1 Department of Chemical and Biomolecular Engineering, University of Delaware, 150 Academy Street, Newark, DE 19716 USA; 2 RAPID Manufacturing Institute and Delaware Energy Institute (DEI), 221 Academy Street, Newark, DE 19716 USA; 3 Department of Chemical Engineering and Applied Chemistry, University of Toronto, 200 College St., Toronto, ON M5S 3E5 Canada

**Keywords:** Computational methods, Theory and computation

## Abstract

A major goal of materials research is the discovery of novel and efficient heterogeneous catalysts for various chemical processes. In such studies, the candidate catalyst material is modeled using tens to thousands of chemical species and elementary reactions. Density Functional Theory (DFT) is widely used to calculate the thermochemistry of these species which might be surface species or gas-phase molecules. The use of an approximate exchange correlation functional in the DFT framework introduces an important source of error in such models. This is especially true in the calculation of gas phase molecules whose thermochemistry is calculated using the same planewave basis set as the rest of the surface mechanism. Unfortunately, the nature and magnitude of these errors is unknown for most practical molecules. Here, we investigate the error in the enthalpy of formation for 1676 gaseous species using two different DFT levels of theory and the ‘ground truth values’ obtained from the NIST database. We featurize molecules using graph theory. We use a regularized algorithm to discover a sparse model of the error and identify important molecular fragments that drive this error. The model is robust to rigorous statistical tests and is used to correct DFT thermochemistry, achieving more than an order of magnitude improvement.

## Introduction

In-silico design of materials involves calculation of ground state properties of materials and molecules from first principles. Density functional theory (DFT) based on the Kohn Sham (KS) formulation of Schrødinger’s equation^1,2^, is widely used for such calculations. While the formulation is theoretically exact, an exact functional is currently lacking^3^. The current implementations of DFT assume an approximate form of the exchange correlation functional that introduces an error in the calculated ground state energies that we refer to as *DFT error*^4^. While approaches such as Δ-machine learning^5^ have been proposed to map thermochemistry between different levels of functional approximations, investigating this error and accounting for it in computational models remains an important active area of research. Such error is particularly important for studying processes where the material is studied in the context of an application that involves surface intermediates connected with gas-phase reactions, products, and byproducts. Materials screening for heterogenous catalysis and surface science are examples of such applications. In such studies, the thermochemistry of the participating gas-phase species is calculated consistently using the same planewave basis sets as for the surface species. Errors in the gas-phase thermochemistry propagate into the surface thermochemistry. Systematic ways to correct such errors are lacking. This is the core motivation for this work and the specific contribution addresses a key fundamental problem in improving the accuracy of materials screening models for surface science and catalysis applications.

One approach to estimate the error in DFT estimates is to evaluate the quantity of interest using several functionals^6^. A similar approach is taken by the Bayesian Error Estimation Functional (BEEF)^7^ and mBEEF^8^ that provide a Bayesian error estimate based on a combination of functionals. Use of multiple functionals is computationally expensive for larger models. Importantly, some of the functionals are correlated and thus, the difference in prediction among functionals does not represent the true error of a functional. Currently, it is not clear how to reduce actual error.

A posteriori correction to the DFT predictions, using a surrogate error model, can enable refinement of predictions. In one approach^9^, the error in the gas-phase free energy was treated as a random variable and error samples were drawn from a probability distribution (a uniform Dirichlet distribution) so that the experimental and model thermochemistry agrees. However, cancellation of errors^10^ and this work indicate that the error is systematic, not random. Nørskov et al.^11^ investigated 11 gas-phase molecules in 21 reactions and assigned an error to the OCO backbone of their molecules. A similar approach based on bond additivity corrections (BAC)^12^ was used by Green and co-workers to fit empirical correction terms for 147 gas-phase species^13^. This was done by regressing the difference in experimental (NIST database) enthalpy of formation and that obtained by coupled cluster calculations. BAC correction terms were proposed exclusively for the G3 level of theory using localized orbitals and the featurization is functional specific. Moreover, the featurization scheme does not consider different bond types. In general, these approaches require prior empirical or expert knowledge to decide the “fragments” to which errors are assigned.

In this work, we investigate first-principles approximation error in calculating the enthalpy of formation of almost 1700 gas molecules. We apply a graph theoretical framework to encode molecules in terms of subgraph frequencies. These subgraphs are molecular fragments, analogous to those in the group additivity framework, pioneered by Benson et al.^14^. We formulate the problem as a subgraph search problem similar to the approach taken by Gu et al.^15^, where this featurization was used for predicting the enthalpy. Here, we use this formalism for learning error in DFT calculated enthalpy. Thus, while Gu et al. used this framework to discover the important groups for estimating enthalpy, we use this framework to discover the important molecular groups for predicting DFT error which might be potentially different from the groups used for predicting the enthalpy itself. The supervised learning algorithm performs two tasks. First, variable selection to reduce the parameter space. Second, regression to predict the error in DFT calculated formation enthalpy. By doing this, it discovers the *important subgraphs* contributing to the error of enthalpy of formation of species in an *automated fashion* and assigns an error to each group. This ‘error model’ is then used to estimate the error for new molecules and correct it. We perform rigorous statistical tests to analyze and interpret our model. We establish a theoretical framework for illustrating the cancellation of DFT error to several tenths of an eV as has been estimated in literature from small scale studies on specific systems^16,17^. We consider different types of atoms and bond types in the molecular structure. An important advantage of this approach is its physical interpretability. Our model is agnostic to the level of theory being investigated and thus we illustrate its application on functionals which are known to be less accurate than the G3 methods used to fit BACs. To our knowledge, this is the first automated model of first principles approximation error in an extensive data set.

In the following, we briefly describe the graph theoretical framework, the LASSO algorithm and the statistical bootstrap tests used for error estimates. We discuss important molecular fragments that drive the error and capture the uncertainty of model results. We then illustrate how to correct DFT calculated formation enthalpy in a post-hoc fashion. We discuss theoretical insights that can be gained from this error framework.

## Methods

Molecules were downloaded from the NIST Chemistry WebBook database^18^ using an automated script which we have made available online (https://github.com/VlachosGroup). The dataset consists of 1676 neutral molecules whose gas-phase enthalpy of formation as well as structures were available. Only molecules comprised of carbon, hydrogen, oxygen, nitrogen, and phosphorus atoms were considered.

### Density functional theory

All DFT calculations were performed using the *Vienna *Ab Initio* Simulation Package* (VASP)^19^. A periodic unit cell of size 20 × 20 × 20 and a 1 × 1 × 1 Monkhorst–Pack mesh for Brillouin zone integration were used. The energy cutoff for the plane wave basis set was 400 eV. A force criterion of 0.01 eV/Å was used for convergence. The ion–electron interactions were represented by the projector-augmented wave (PAW)^20^ method. Two exchange correlation functionals were considered: the Perdew–Burke–Ernzerhof (referred to henceforth as the ‘PBE functional’) of the Generalized Gradient Approximation (GGA) level of theory^21^ and the Local Density Approximation (LDA)^2,22^. In this work, we focus on planewave basis set since these are used in the surface science and catalytic reaction mechanisms that involve surface and gas species, as mentioned in the introduction.

### Thermochemistry

All thermochemistry was calculated using the pMuTT Python package^23^. The vibrational modes were treated as simple harmonic oscillators and zero-point corrections were applied using the pMuTT package. All species were treated as ideal gases at the same conditions as the experimental values (298.15 K and 1 atm). The values were corrected to account for the difference of formation enthalpy of the atoms due to the choice of reference as has been described in literature (Eq. 1 of Ref.^24^).

### Graph theoretical framework

For this work, we utilize a graph theoretical framework to featurize molecules. A molecule is defined as a “master graph” whose nodes and edges represent the nuclei and bonds, respectively. For the sake of computational efficiency, all hydrogens are lumped and not treated explicitly as nodes. Thus, each node is identified as a carbon, oxygen, nitrogen, or phosphorus atom. Similarly, each edge represents a single, double, or triple bond. The unique subgraphs of a master graph are the descriptors of the molecule. A data matrix for the entire set is constructed where each column represents a unique subgraph, and each row represents a molecule. The element *d*_*ij*_ of the data matrix corresponds to the frequency of subgraph *j* in molecule *i,* i.e., how many times subgraph *j* is found in molecule *i*. It is to be noted that the descriptors are strictly geometrical and 2 dimensional. Furthermore, we mine all subgraphs in an automated fashion without biasing the results using prior knowledge. We find all subgraphs up to a maximum size of 4 linkages to be suitable. The package RDKIT^25^ was used to represent molecules as graphs and NetworkX^26^ was used to mine unique subgraphs up to isomorphism, ensuring rotational and translational symmetry. All plotting was done with the Matplotlib^27^ Python package.

### Statistical modeling

To systematically discover the subgraphs contributing to the error, we use the LASSO (least absolute shrinkage and selection operator)^28^ algorithm. LASSO performs descriptor (here subgraph) selection by considering all possible subgraphs up to a certain size and selecting the ones that minimize the loss function. The loss function of LASSO is formally:

1 $$L_{\lambda } \left( {a,\user2{w}} \right) = \frac{1}{2}a^{2} + \lambda \left| {\left| \user2{w} \right|} \right|_{1}$$

where $~a = y_{{predicted}} - y_{{true}}$ and $y_{{true}}$ is any response of interest, λ is a hyperparameter controlling the model sparsity estimated using tenfold cross validation on the training data, and $\left| {\left| \user2{w} \right|} \right|_{1}$ is the L1 norm of the parameter vector ***w***. λ is the regularization parameter. When λ = 0, the problem is a traditional least squares regression. By increasing λ, more parameters are set to zero, leading to a sparser model with less descriptors. When λ is too large, the model is not as accurate as it uses too few descriptors. Consequently, there is an optimum λ that strikes a balance between model simplicity and accuracy. This λ is the optimum of the bias-variance tradeoff. The response here is the (experimental-DFT enthalpy of formation).

The data set of 1676 points was split randomly into a training (90%) and an external (not seen by the model during training) test-set (10%). The training set was repeatedly subsampled (with replacement) by splitting it in 60–40 ratios. The 60% sample was used to train the model and the remaining 40% (validation set) for calculation of performance metrics, such as mean absolute error (MAE) and the subgraph coefficients selected by LASSO. This process was repeated 1000 times to give a population of these metrics and capture the uncertainty and performance of the model. Note that the externally held out test set is fixed and thus cannot be sampled from.

*P*($\epsilon$ < *2*) is a probabilistic measure of model accuracy that we introduce. It represents the probability that the model predicts within 2 kcal/mol (absolute) for a randomly chosen molecule and serves as an empirical surrogate to the posterior probability of the model performing within 2 kcal/mol accuracy. As such, the threshold of 2 kcal/mol is derived from pragmatic considerations. However, it is straightforward to apply the definition to any user-defined threshold accuracy.

*P*($\epsilon$ < 2) is estimated as:

2 $$P\left( {\epsilon < 2} \right) = \frac{1}{N}\mathop \sum \limits_{i}^{N} H\left[ {2 - \epsilon _{i} } \right]$$

where N is the total number of points in the dataset, H[n] is the Heaviside step function (= 0 for n < 0, 1 otherwise) and $~\epsilon _{i}$ is the absolute error in the model prediction for point *i.* In Eq. (2), we run the sum over all molecules for which the absolute error is greater than 2 kcal/mol. This is done to estimate the posterior probability of the model performing within 2 kcal/mol accuracy. In a real application, we would expect most molecules to be drawn from near the mode of this distribution. Hence, instead of estimating for a given random molecule what the model error is likely to be (which is what metrics such as MAE try to estimate), this metric tells us how likely the model is to fail catastrophically (as defined by the model predicting with an accuracy worse than the threshold accuracy of 2 kcal/mol).

## Results and discussion

### Distribution of errors

**Figure 1:**
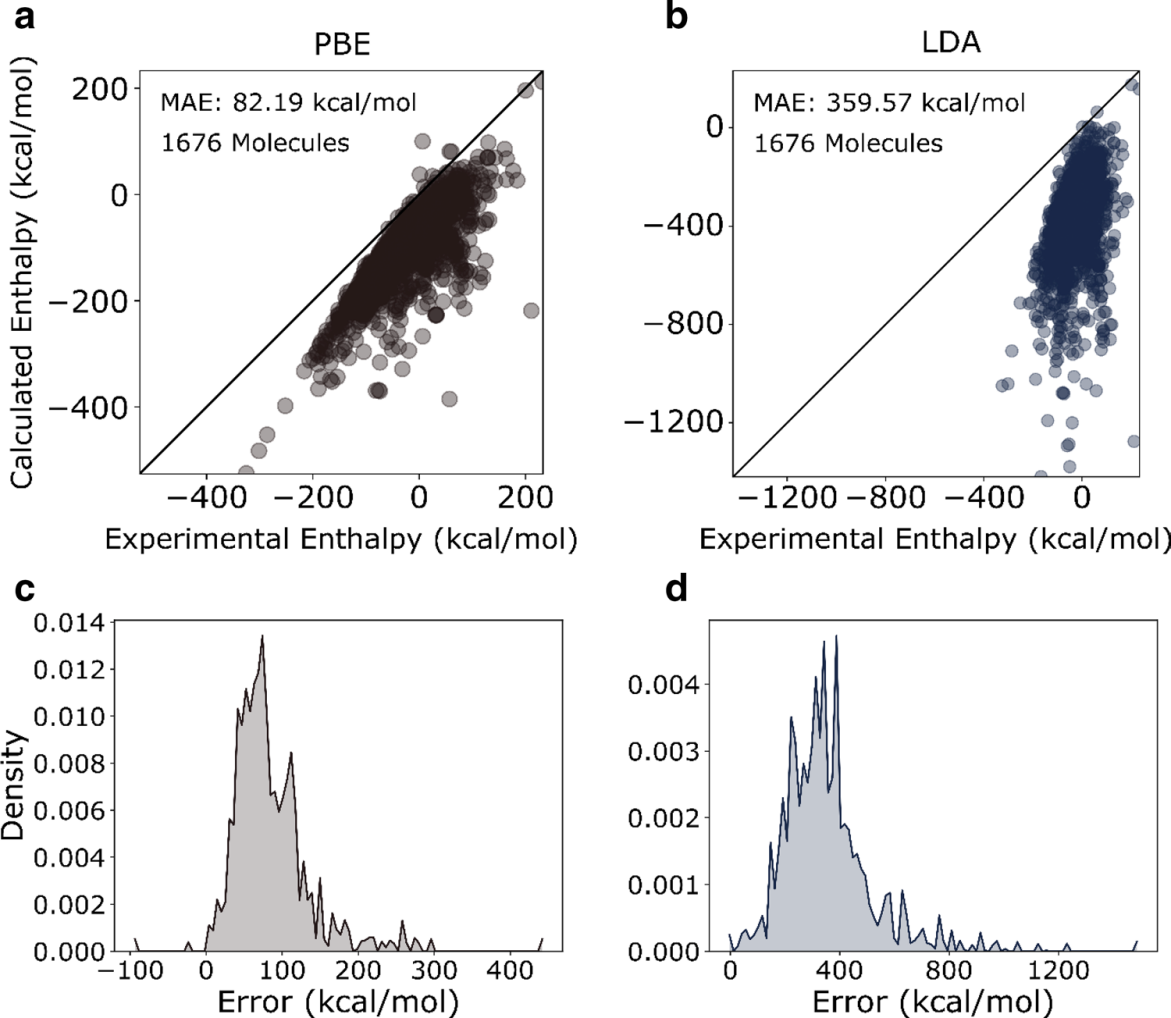
Parity plot of calculated formation enthalpy for 1676 gas species using the (**a**) PBE and the (**b**) LDA functional at 298.15 K and 1 atm versus experimental enthalpies of formation. Population density of errors in enthalpy of formation (298.15 K, 1 atm) using the (**c**) PBE and (**d**) LDA functional.

The parity plot of calculated versus the NIST formation enthalpy values (Fig. 1a, b) suggests a systematic bias by both functionals. The PBE functional shows a smaller error (MAE: 82.19 kcal/mol) compared to the LDA functional (MAE: 359.57 kcal/mol) consistent with the level of theory. The population density of errors in Fig. 1c, d show a unimodal distribution of error with non-zero means underscoring systematic under-prediction.

### Linear models of the error

**Figure 2:**
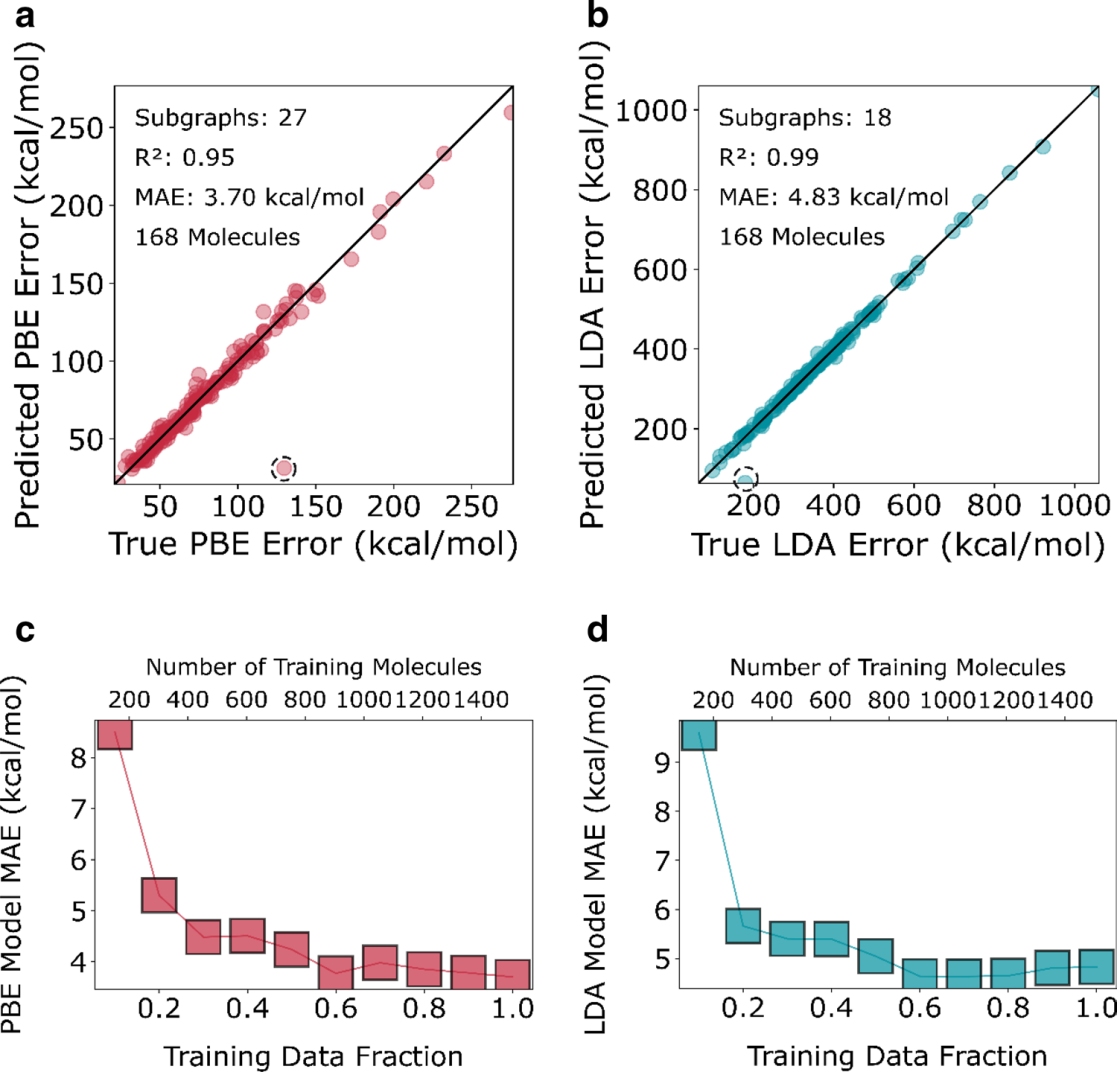
Parity plot of error formation enthalpy predicted by surrogate LASSO model on the test set versus the true error in enthalpy calculated using the PBE functional. The outlier circled is phosphorus dioxide (129.7, 31.25). (**b**) Error in enthalpy calculated using the LDA functional. The circled outlier is again phosphorus dioxide (179.6, 64.47). Learning curves for the (**c**) PBE and (**d**) LDA models. The boxes represent mean absolute error on the externally held out test set on models trained on the respective fractions of the training set.

The performance of the error model on the external test set is shown for both functionals in Fig. 2a, b. Out of 713 subgraph descriptors, the LASSO algorithm identifies *27* subgraphs for the PBE error model with which it can predict error within an accuracy of 3.7 kcal/mol and an R^2^ value of 0.95. The LDA model similarly identifies 18 subgraphs and predicts error to 4.83 kcal/mol with an R^2^ value of 0.99.

A significant outlier is phosphorus dioxide (circled in Fig. 2a, b) due to the inability of the models to capture the physics of this particular molecule. The entire dataset of 1676 molecules has only 5 molecules with a phosphorus atom and only this molecule contains the $\dot{P}O$ 2 motif. Thus, it is likely that with more training samples of this linkage, the model would learn the error (further discussion is given in section S5 of the Supplementary Information).

The learning curves of the LDA and the PBE error models are shown in Fig. 2c, d, respectively. These curves are generated by training models on increasing fractions of the training data and recording the MAE on the test set. 60% of the training data (~ 900 molecules) is sufficient to train the models, as indicated by the plateau of the MAE at the 0.6 mark in the learning curves.

**Figure 3:**
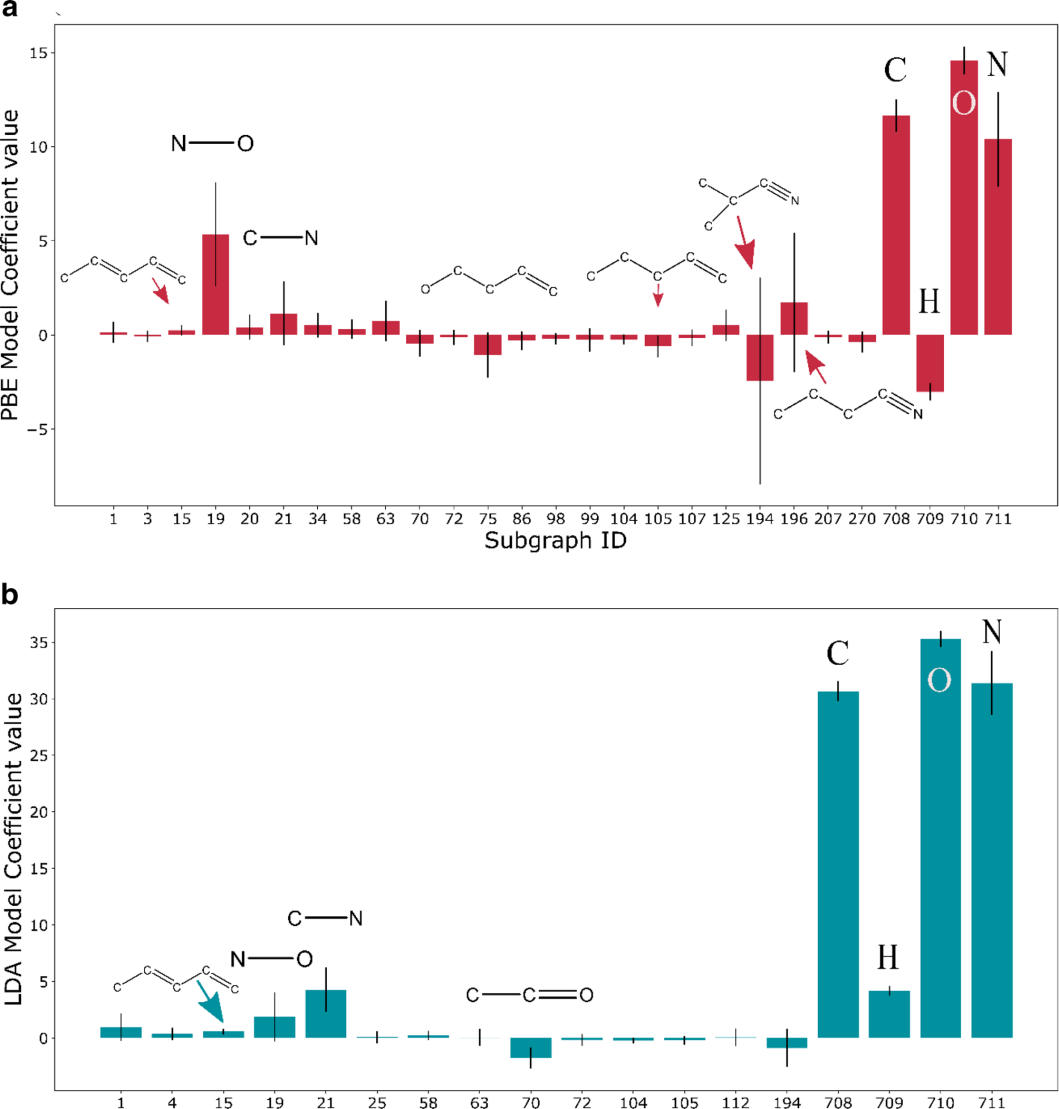
Coefficients of the key groups identified by the (**a**) PBE and (**b**) LDA error model. A list of all groups has been made available with the Supplementary Materials and their coefficients can be found on the GitHub page.

Further insight is gained from the coefficients of the various groups contributing to the error model (Fig. 3) of all groups has been made available with the Supplementary Materials and their coefficients can be found on the GitHub page. For the PBE model (Fig. 3a), the major contributors are zeroth-order subgraphs of atoms of carbon (Group 708, 11.65), hydrogen (Group 709, − 3.01), oxygen (Group 710, 14.59), and nitrogen (Group 711, 10.39), all in kcal/mol. There are significant contributions from two atom groups of N–O (Group 19, 5.35) and C–N (Group 21, 1.15). We call this first-order contributions to the error and can be interpreted as errors in calculating bond enthalpies. Finally, there are higher order contributions such as branched (Group 194, − 2.44) and linear (Group 196, 1.72) cyanide fragments and C–C=C–C=C (Group 15, 0.25) that indicates the presence of an aromatic ring. Explicit identification of the aromatic ring and other higher order structures is not possible due to the subgraph size to 4 linkages. Another interesting fragment is the carbonyl fragment C–C=O (Group 70, − 0.44). Similar trends for the LDA functional are shown in Fig. 3b. Interestingly, the contribution from the hydrogen atom has opposite sign between the two models. The PBE functional overpredicts the enthalpy of formation of Hydrogen atom whereas the LDA functional underpredicts it. The distributions of the coefficient of the H atom, obtained over 1000 samples, do not overlap (Figure S3) suggesting the different sign is not an artifact of the fitting. Since the error is defined as (experimental-DFT enthalpy of formation), a positive value of a coefficient for a group means that for each occurrence of that group in the molecules, the model predicts that the error is positive i.e., the DFT calculated enthalpy of formation is lower than the experimental value. The converse is true for negative coefficients.

The error bars in the coefficients are obtained by bootstrap simulation (at the 95% confidence interval). The spread of the coefficients indicates multiple families of solutions typical of poorly determined parameters. To fit 714 parameters there are ~ 900 training points in each run. Moreover, since LASSO converges to a sparse solution using gradient descent, there might be multiple families of such sparse solutions. This is further complicated by the fact that molecular fragments are often linearly dependent (e.g., C–C=C can be expressed as C–C + C=C). Thus, it is not straightforward to use the error bars to comment on the significance of individual parameters. For this reason, we introduce the normalized model frequency of each “active” coefficient (i.e., of the groups chosen by the final model that are depicted in Fig. 3) across the bootstrap simulation. This is defined as:

3 $$Normalized\;~model~\;frequency~\;of\;~coefficient~\;w_{j} = \frac{1}{N}\mathop \sum \limits_{i}^{N} I\left[ {\left| {\left( {w_{{j,i}} } \right)} \right| > 0} \right]$$

**Figure 4:**
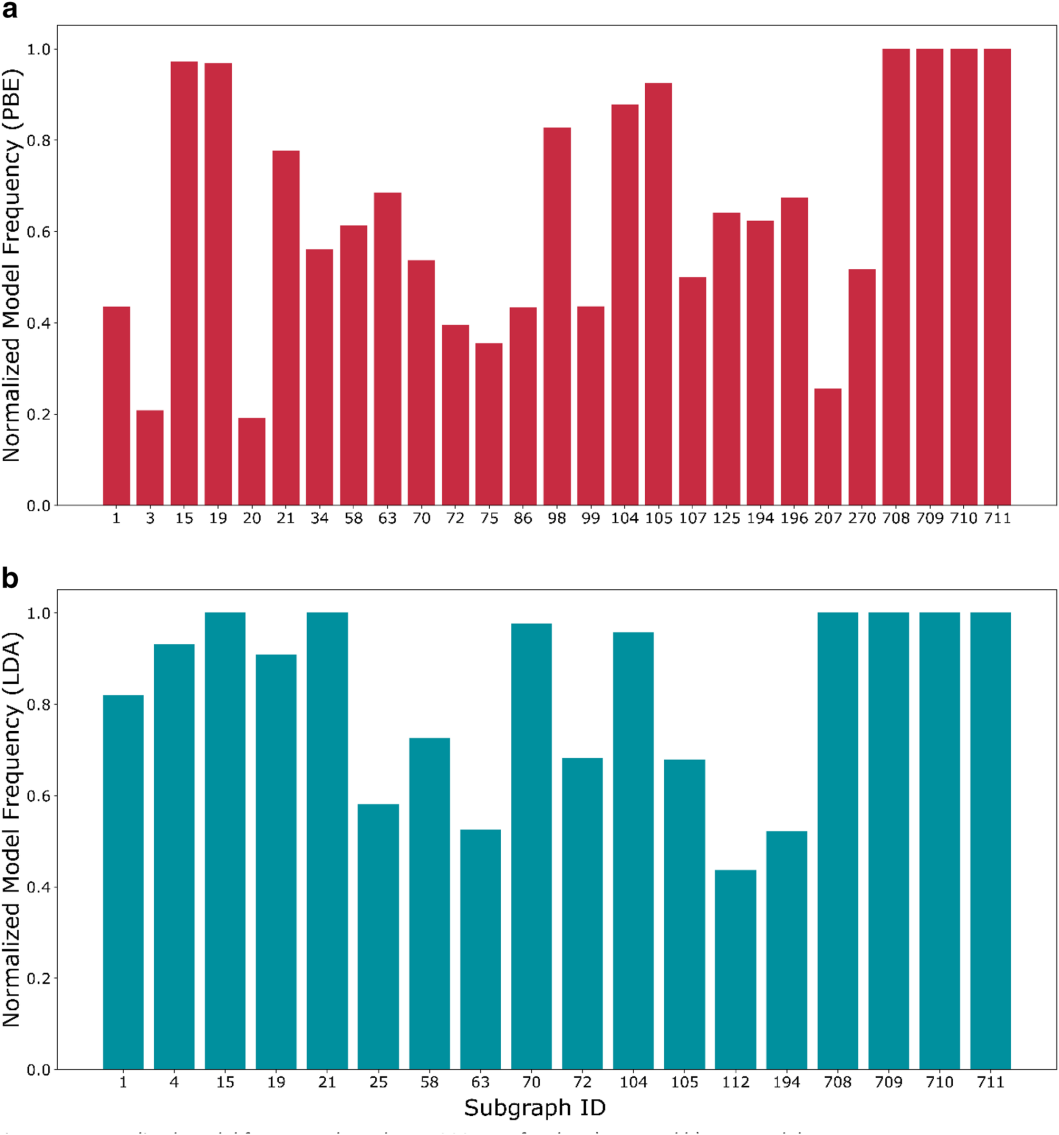
Normalized model frequency based on 1000 runs for the (**a**) PBE and (**b**) LDA models.

Here I(condition) is the indicator function which is 1 (0) if the condition is true (false). Abs() is the absolute function and ${w}_{j,i}$ indicates the coefficient ${w}_{j}$ in the ith simulation. A normalized model frequency close to 1 indicates that the coefficient ${w}_{j}$ is chosen by LASSO in most simulation runs. These are plotted for both models in Fig. 4. The high values for most coefficients indicate that they are all important for calculating the error. The exact numerical values may vary based on the (degenerate) family of solutions chosen by LASSO. The low normalized model frequency of some groups, e.g., 3, 20 and 207 in the PBE error model (Fig. 4a), are associated with groups with low coefficients and probably indicate “noisy” groups that do not affect the error. The full spectrum mined for both the models is in Figure S2.

**Figure 5:**
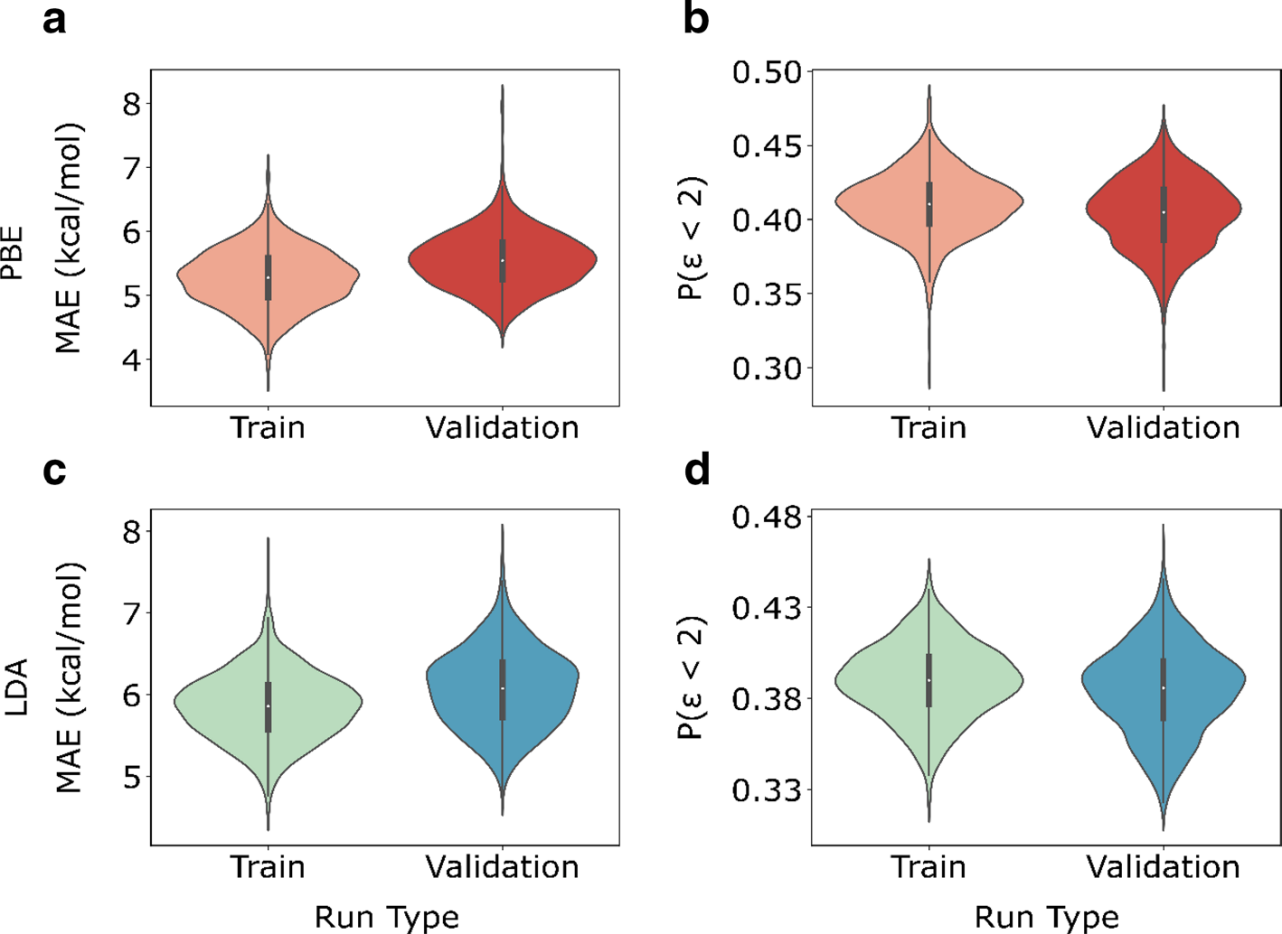
Violin plots of train and validation MAE during bootstrap simulations of the (**a**) PBE and (**c**) LDA model. Violin plots of train and validation P ($\epsilon$ < 2) during bootstrap simulations of the (**b**) PBE and (**d**) LDA model.

While interpreting individual coefficients might be complex, we used the bootstrap simulations to generate a population of model MAE and *P*($\epsilon$< 2) values to guarantee the performance of the general framework. The violin plots of the generated distributions are plotted in Fig. 5 for both models and metrics. Note that a violin plot is a boxplot with the surrounding shading representing the density of points i.e., at a given y-axis value, the width of the violin plot is proportional to the number of points with that y-axis value. From Fig. 5a, c, the median MAE on the validation for the PBE error model is around 5.5 kcal/mol and for the LDA error model is around 6 kcal/mol. The long tail of the PBE error model MAE distribution is to be noted. We believe that this tail corresponds to runs with stratified partitioning with the testing set having bigger molecules and different subgraph distributions than the training set. This is discussed in detail in section S4 of the Supplementary Information. In general, model results above are optimistic and greater MAE in the wider chemical space should be expected. From Fig. 5b, d, both the PBE and LDA error models can be expected to be accurate to within 2 kcal/mol about 40% of the time.

## Discussion

In this work, important subgraphs driving the error in formation enthalpy calculated using the PBE and LDA functionals were discovered in a data-driven fashion. This has important implications for studying the propagation of errors to the enthalpy of an elementary reaction (or any reaction in general).

Firstly, the dominant contributions seem to be from zeroth order atomic contributions. This could be a manifestation of the unsuitability of plane wave DFT calculations for isolated atom thermochemistry (which is required for referencing the enthalpies of formation) or the insufficient cancellation of errors between the different phases of bonded molecules and their atomic components, as discussed in^29^. Using just the zeroth and first order (atom–atom bonds) subgraphs, it is possible to make a predictive model with an MAE of 4.15 kcal/mol (Figure S6). This corresponds to the BAC correction model which was proposed for G3 level functionals with localized orbitals. In this case, similar corrections are discovered in a data-driven fashion for the PBE and LDA functionals with planewave basis sets. Moreover, higher level subgraph contributions account for the further ~ 0.5 kcal/mol increase in accuracy of the full model.

Since the number and types of atoms are the same on the reactant and product side of a reaction, additive corrections associated with the enthalpies of specific atoms cancel out exactly due to sign inversion between reactants and products. Thus, the dominant zeroth-order groups cancel out and do not contribute to the error in the calculation of the overall enthalpy of reaction. Molecules having similar number of these groups (including similar atoms) will have similar error in enthalpies of formation. A rigorous study of this phenomena is shown in section S2 of the Supplementary Information. However, higher-order error contributions might be manifested in reaction thermochemistry, if the reaction involves breaking or forming any of the important subgraphs driving the DFT error. For example, when a reaction involves breaking or forming C–N or N–O bonds, the reaction thermochemistry would exhibit considerable error. In such cases, the cancellation of error heuristic would fail even if the reactants and products resemble each other in all other ways. If we consider that the error in reaction enthalpy is the major contributor to the reaction free energy error, these errors get exponentially amplified in the calculation of equilibrium constants (K = ${e}^{\frac{-\Delta {G}^{0}}{RT}}$). Clearly, the error propagated to the reaction enthalpy is reaction specific. While it would be possible to make a separate model of errors in reaction enthalpies, the space of reactions is vast. Importantly, the groups identified here represent fundamental corrections and thus gives a way of breaking down and explaining reaction enthalpies in terms of fragments of participating species. This is due to the simple fact that a chemical reaction is a linear combination of species and thermochemistry (enthalpy, entropy, free energy) has additive character due to being a state variable.

Due to the linear additive nature of the groups, the model, in general, will predict a larger DFT error for bigger molecules (having more groups) than smaller molecules. This property of the model agrees with the error behavior observed empirically. Figure S4a and b show the roughly linear dependence between molecule size (defined as the total number of atoms in the molecule) and error in formation enthalpy calculated using both the functionals. This assumes that groups occur independent of each other. While this is obviously not true strictly (bigger groups can be considered as linear combinations of smaller groups), this assumption roughly holds for the groups selected by LASSO. This is illustrated in Figure S5a and b, by plotting the heatmap of the absolute value (correlation and anti-correlation is treated equally) of the Pearson coefficient of correlation between the groups chosen by LASSO for both the functionals. The small off diagonal terms in the heatmap indicate weak correlation between the groups. This also depends on the maximum subgraph size cutoff. The size of groups (subgraphs) will increase with this cutoff but since bigger groups tend to be linear combinations of smaller ones, this will cause increasing correlation between the descriptors.

Finally, the parity plots in Fig. 2a, b show that the residual errors of the LASSO DFT error models are nearly random with no apparent structure around the diagonal. We think that this represents the residual “random” error implicit in the choice of DFT functional i.e., the random component of DFT error which remains after the systematic component is explicitly accounted for (via subgraph frequency-based error correction). The mean absolute value of this residual error is 4–5 kcal for both the functionals, shown in consistent with the order of magnitude estimates of DFT enthalpy error (with error cancellation) of few-tenths of an eV^16,17^.

### Correcting the enthalpies of formation of species

**Figure 6:**
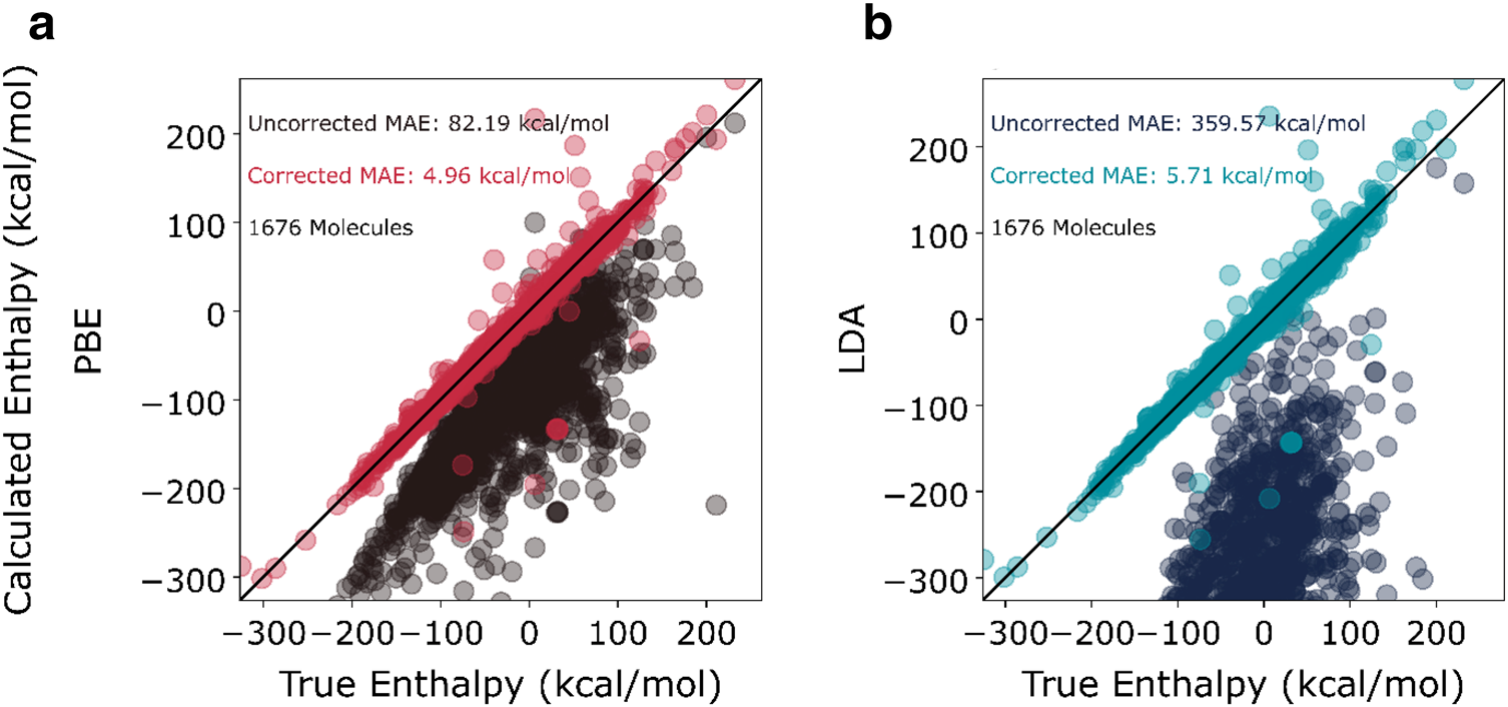
Performance of the surrogate LASSO error model in correcting enthalpies using the (**a**) PBE and (**b**) LDA for the entire dataset.

An important application of the approximation error is that it enables a posteriori correction of the calculated thermochemical properties. This is illustrated as a proof of concept using the error model to correct the entire dataset. Note that this is the model trained on 90% of the data (training set) and used to predict the error of the entire data set. The dark blue and black points in Fig. 6a, b (PBE and LDA respectively) indicate the uncorrected gas-phase enthalpies of formation. The red (Fig. 6a) and green–blue (Fig. 6b) points indicate the calculated values corrected using the LASSO error model. It is evident that the a posteriori correction achieves greater than an order of magnitude and almost two orders of magnitude improvement for the PBE and LDA functional, respectively.

## Conclusions

We have investigated the DFT errors in calculating the formation enthalpy of gas molecules. The errors are systematic for both LDA and GGA (PBE). We have proposed a space of molecular graphs to study first principles approximation errors. We show that the errors are well described by 4-edge fragments of the molecules and can be predicted by the LASSO regressor using a relatively small number of 27 and 18 subgraphs for PBE and LDA, respectively. These fragments were mined automatically from the high dimensional subgraph space. The chief contributions to the error are from atoms (zeroth order contributions) with significant contributions from select single bonds (first order or bond contributions) and a few higher order contributions from multiple bond groups. This framework was used to explain cancellation of errors of DFT energies and provide a diagnostic tool to identify when the assumption of error cancellation fails. This model could increase the accuracy of the GGA (LDA) calculation by more than an order of magnitude (almost two orders of magnitude).

## Supplementary Information

Supplementary Information.
