# Supplementary material for: Regularized machine learning on molecular graph model explains systematic error in DFT enthalpies
Source: Sci Rep. 2021 Jul 13;11:14372. doi: 10.1038/s41598-021-93854-w (PMC8277863; doi:10.1038/s41598-021-93854-w)
Supplement: Supplementary file 1 — Supplementary Information. [file 41598_2021_93854_MOESM1_ESM.docx]

**Supplementary Information**

**Regularized Machine Learning on Molecular Graph Model Explains Systematic Error in DFT Enthalpies**

Himaghna Bhattacharjee^a,b^, Nikolaos Anesiadis^c^, Dionisios G. Vlachos^a,b*^

^a^Department of Chemical and Biomolecular Engineering, University of Delaware, 150 Academy Street, Newark, Delaware 19716, United States

^b^RAPID Manufacturing Institute and Delaware Energy Institute (DEI), 221 Academy Street, Newark, Delaware 19716, United States

^c^Department of Chemical Engineering and Applied Chemistry, University of Toronto, 200 College St., Toronto, ON M5S 3E5, Canada

^*^Corresponding author: [vlachos@udel.edu](mailto:vlachos@udel.edu) (D.G. Vlachos)

# S1. Correlation Matrix

The correlation matrix is used to represent the correlation between responses. To compute the variable correlation matrix, we begin by defining a data matrix for the data set consisting of ***p*** variables and ***n*** observations:

$\boldsymbol{X}= \left[ \begin{matrix} x_{1, 1} & \cdots& x_{1,p} \\ \vdots& \ddots& \vdots\\ x_{n,1} & \cdots& x_{n, p} \end{matrix} \right]$ (1.1)

For this data matrix, we can define a covariance matrix:

$\boldsymbol{S}= \left[ \begin{matrix} s_{1}^{2} & s_{1,2} & \begin{matrix} s_{1,3} & \cdots& s_{1,p} \end{matrix} \\ s_{2,1} & s_{2}^{2} & \begin{matrix} s_{2,1} & \cdots& s_{2,p} \end{matrix} \\ \begin{matrix} s_{3,1} \\ \vdots\\ s_{p,1} \end{matrix} & \begin{matrix} s_{3,2} \\ \vdots\\ \ldots\end{matrix} & \begin{matrix} \begin{matrix} s_{3}^{2} & \cdots& s_{3,p} \end{matrix} \\ \begin{matrix} \vdots& \ddots& \vdots\end{matrix} \\ \begin{matrix} \ldots& \ldots& s_{p}^{2} \end{matrix} \end{matrix} \end{matrix} \right]$ (1.2)

where,

$s_{j}^{2}= \frac{\sum_{i=1}^{n} {(x_{i,j}- \bar{x_{j}})}^{2}}{n}$ is the variance of the j^th^ variable,

$s_{j,k}= \frac{\sum_{i=1}^{n} (x_{i,j}- \bar{x_{j}})(x_{i,k}- \bar{x_{k}})}{n}$ is the co-variance between the j^th^ and k^th^ variables,

$\bar{x_{j}} = \frac{\sum_{i=1}^{n} x_{i,j}}{n}$ is the average of the variable *x_j_* over all the observations.

Finally, the correlation matrix is:

$$\boldsymbol{R}= \left[ \begin{matrix} 1 & r_{1,2} & \begin{matrix} r_{1,3} & \cdots& r_{1,p} \end{matrix} \\ r_{2,1} & 1 & \begin{matrix} r_{2,1} & \cdots& r_{2,p} \end{matrix} \\ \begin{matrix} r_{3,1} \\ \vdots\\ r_{p,1} \end{matrix} & \begin{matrix} r_{3,2} \\ \vdots\\ \ldots\end{matrix} & \begin{matrix} \begin{matrix} 1 & \cdots& r_{3,p} \end{matrix} \\ \begin{matrix} \vdots& \ddots& \vdots\end{matrix} \\ \begin{matrix} \ldots& \ldots& 1 \end{matrix} \end{matrix} \end{matrix} \right](1.3)$$

where,

$r_{j,k}= \frac{s_{j,k}}{s_{j}s_{k}}= \frac{\sum_{i=1}^{n} (x_{i,j}- \bar{x_{j}})(x_{i,k}- \bar{x_{k}})}{\sqrt{\sum_{i=1}^{n} {(x_{i,j}- \bar{x_{j}})}^{2}} \sqrt{\sum_{i=1}^{n} {(x_{i,k}- \bar{x_{k}})}^{2}}}$ is the Pearson correlation coefficient between variables j and k.

Note that the Pearson correlation coefficient varies from -1 (perfect anti-correlation) to 0 (no correlation/orthogonal) to 1 (perfect correlation).

## S2. Error Cancellation

Error cancellation is well-known and arises from correlations. The errors of similar molecules, being similar, cancel one another out. Such a behavior might be expected for example between ethane and propane. To understand correlations, “similarity” needs to be formalized. We propose that since the subgraph frequency space is based on the notion of groups, closeness in this space can be used as a measure of chemical similarity, i.e., molecules that resemble each other chemically are located close to each other in the subgraph frequency feature space. We use cosine similarity, which is a measure of geometric collinearity, to identify nearest neighbors in the subgraph space and compare their DFT errors.

In Figure S1, the errors of ‘similar’ molecules are plotted as a parity plot for both functionals. The errors are correlated for neighbors in subgraph frequency space with correlation coefficient of 0.82 for the PBE functional and 0.83 for the LDA functional. This suggest a strong degree of correlation and might explain why calculations of overall reaction thermochemistry are more accurate than that of individual molecules. The large mean absolute error of ~82 kcal/mol (for PBE) and ~360 kcal/mol (for LDA) does not manifest as severely in DFT models because the parameters involve differences in the thermochemistry of similar molecules (reactants and transition states for rate constants and reactants and products for reaction thermochemistry) instead of absolute values. Note that this analysis is exploratory and does not require a specific data-driven predictive model.


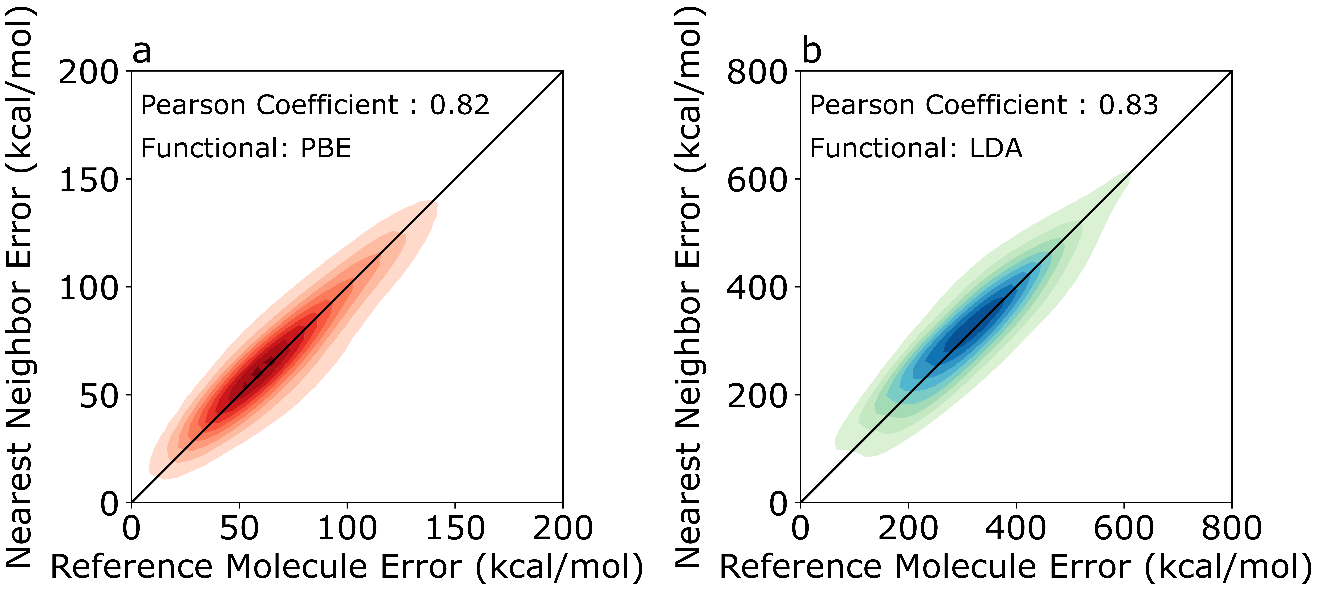


Figure S1. Error in calculated enthalpy for pairs of molecules having the highest cosine similarity in the subgraph space, referred to as nearest neighbors. (a): PBE functional. The Pearson coefficient of correlation between error for similar molecules is 0.82 with a 2-sided p-value ~0. (b): LDA functional. The Pearson coefficient of correlation between error for similar molecules is 0.83 with a 2-sided p-value ~0.

# S3. Plots


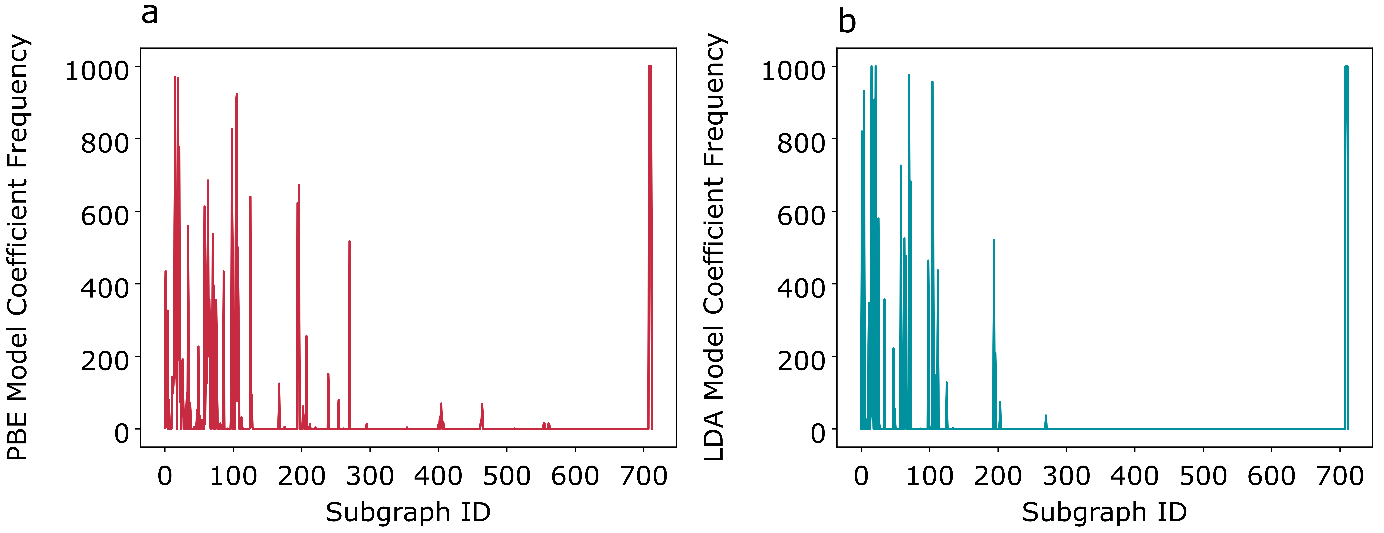


Figure S2. Coefficient frequency of all groups for a) the PBE model and b) the LDA model. Note that these are not normalized.


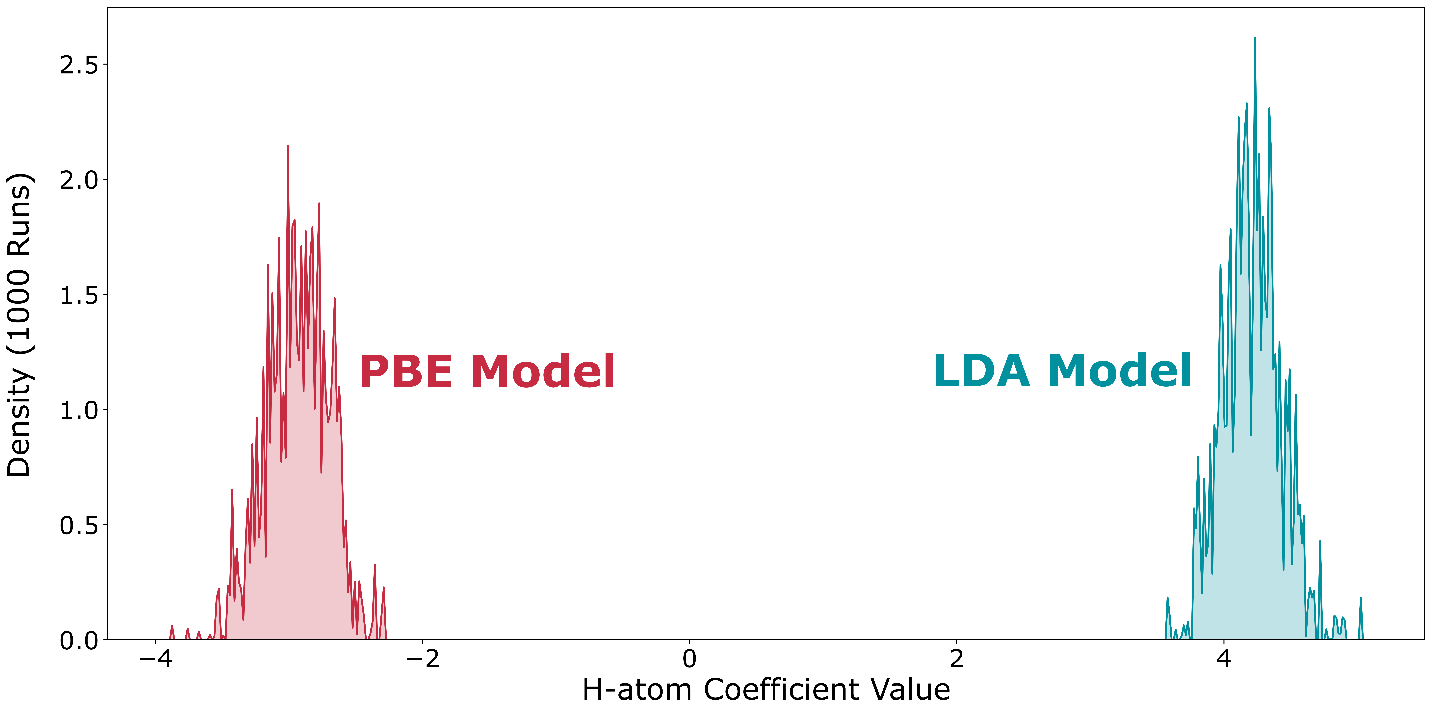


Figure S3. Distribution of H-atom coefficients for the PBE and LDA Models obtained by a bootstrap run consisting of 1,000 runs.


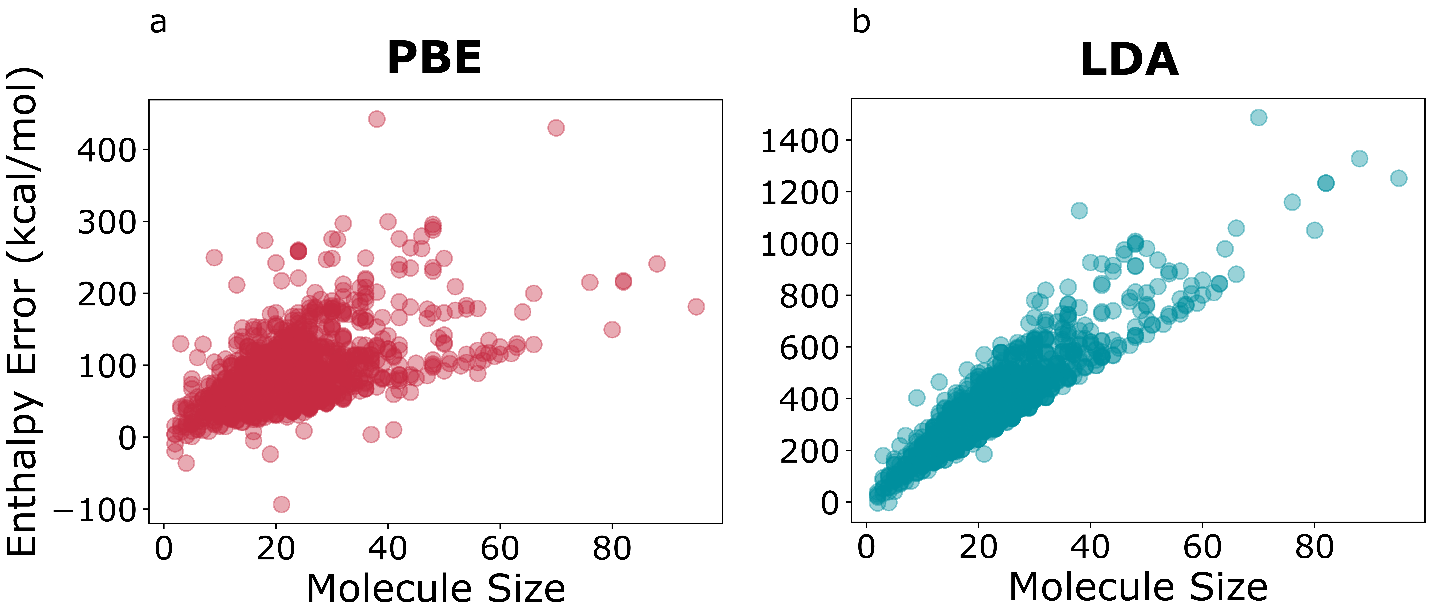


Figure S4. Error in enthalpy calculated using the a) PBE and b) LDA functional as a function of molecular size, defined as the total number of atoms in the molecule. Notice the roughly linear growth of error with molecule size. These plots for the entire dataset of 1,676 molecules.


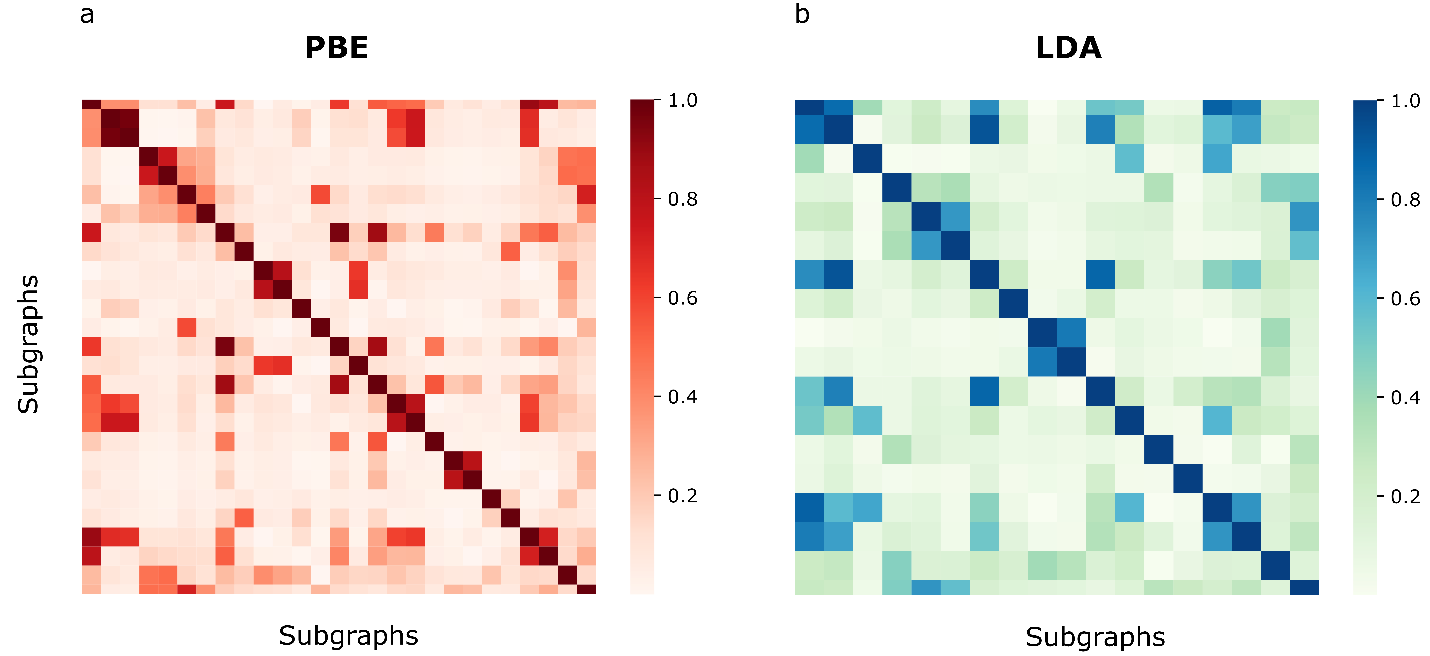


Figure S5. Correlation matrix heatmap showing the absolute Pearson correlation between the subgraph descriptors chosen by the a) PBE and b) LDA model. The heatmap is plotted for the entire dataset of 1,676 molecules.


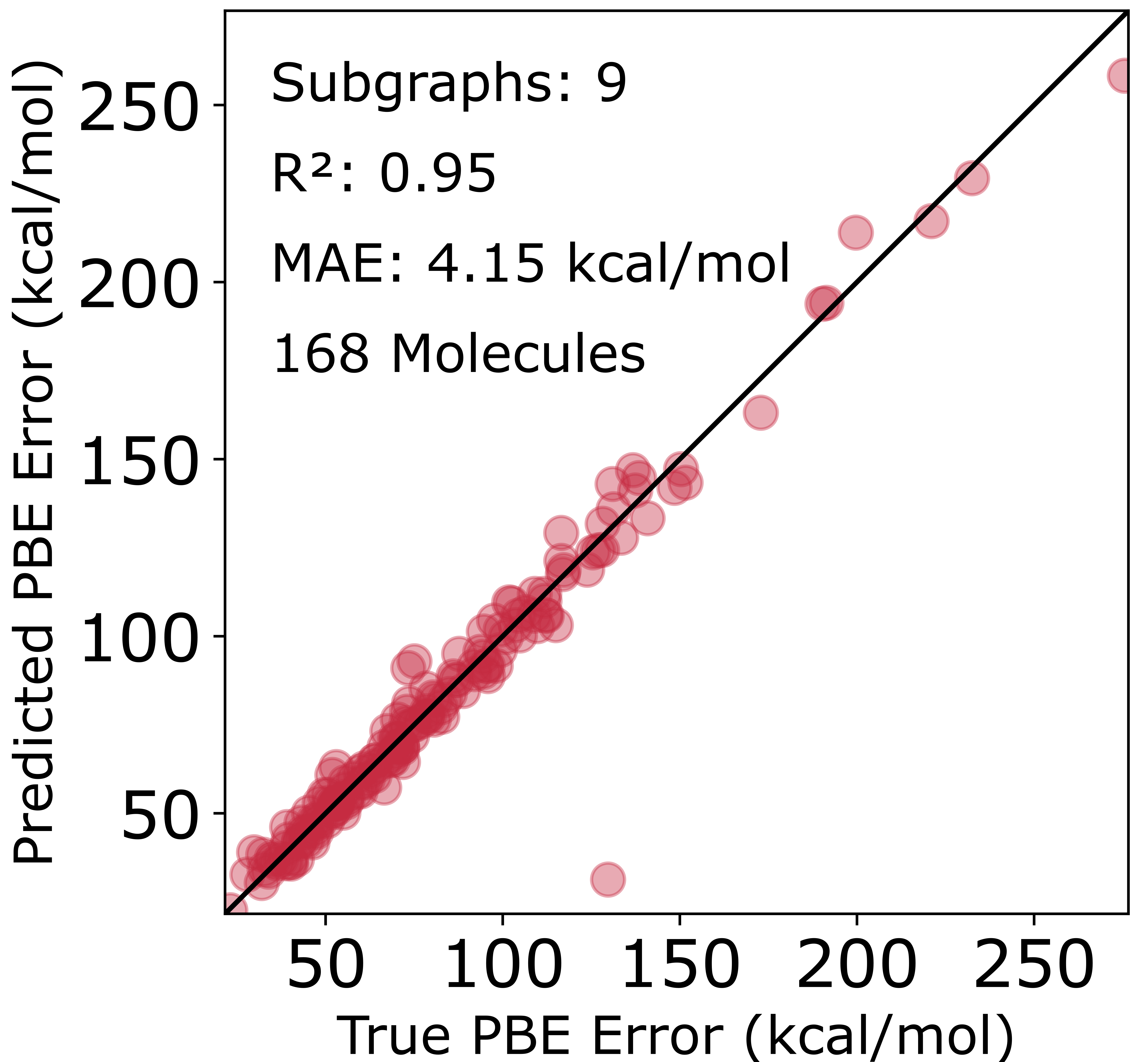


Figure S6. Parity plot of PBE error model trained on only 0 (atomic) and first order (atom-atom bond) subgraphs.

# S4. Molecule Size Effects

To investigate the effect of molecule size, we first verified that the model residuals during training and testing are independent of molecule size (defined as the total atoms in a molecules). The residuals are defined as *predicted response – true response*. The scatter plots of the PBE model residuals during training and testing are shown in Figure S7. The scatter shows no apparent dependence on molecule size.


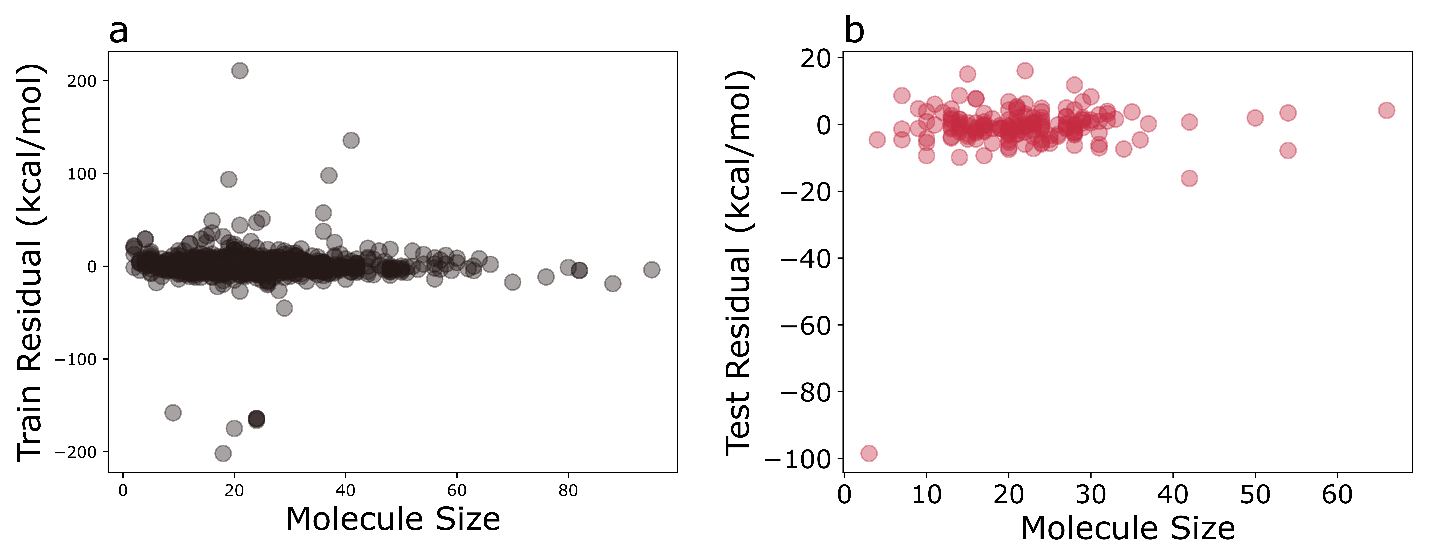


Figure S7. Model residuals vs Molecule Size during a) Training and b) Testing. Note the absence of any observable correlation inn the scatter. The residuals are defined as (predicted – true) response.

However, on doing a 90 / 10 (train / test) stratified split based on molecules size, with larger molecules in test set, we find a high test MAE of 8.24 kcal/mol for the PBE model as shown in the parity plot of Figure S8. This corresponds to the long tail in the testing MAE violin plot in Figure 5a. This deterioration of model performance is probably due to the structural difference between the subgraphs in the training and testing set when the split is stratified. In Figure S9 we look at the distribution of subgraphs in the training and test dataset when the split is done in a random fashion and in a stratified fashion. The metric we use on the y-axis is normalized subgraph frequency. For a particular subgraph, this is the number of times the subgraph occurs in the dataset normalized by the number of molecules in the dataset. For clarity, only the important subgraphs chosen by LASSO is considered. In the random split, the distribution is unchanged between training and testing. This translates to a better model performance on the test set. In the stratified split however, the distribution changes dramatically between training and testing. This makes it difficult for the model to make as accurate a prediction as the random split. We believe that the current investigation spans the chemical set of interest to the study of most chemistries. Thus, a stratified split does not represent a practical use case. However, if there does arise a need to predict much larger molecules than what was used to train the model, we would expect the model accuracy to deteriorate, a feature typical of most data-driven approaches in the extrapolative regime. In such a case, it might be helpful to introduce some of the bigger molecules in the training set and refine the model parameters.


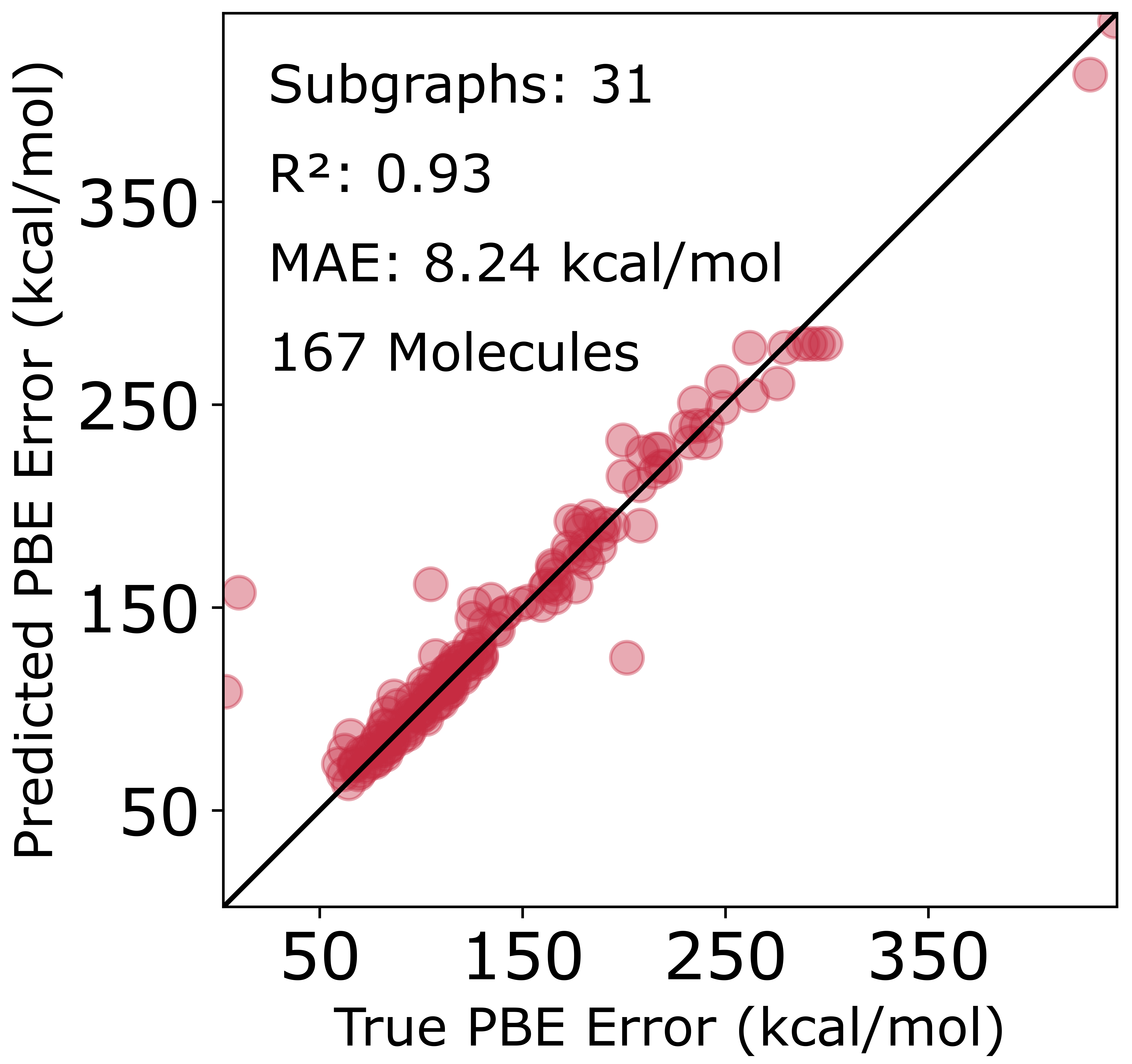


Figure S8. Parity plot of predicted PBE error to true PBE error when the 90 / 10 train / test split is conducted in a stratified fashion with bigger molecules in the test set.





Figure S9. Normalized Subgraph Frequency for training and test set when the 90 / 10 train/ test split is dome randomly and when it is done in stratified fashion. The subgraphs shown are the important subgraphs chosen by the PBE LASSO model in the main text.

# S5. Distribution of Elements in the Dataset

An analysis of the LASSO models (Figure 2a-b) suggests that the most severe outlier of the model predictions is the molecule with the $PO$2 motif. We believe that this failure is due to the sparsity of this group in the dataset. The bar plot in Figure S10 plots the frequency of elements in the training and test datasets or the number of atoms corresponding to each element in these datasets. Compared to other elements, phosphorus is very sparse. The outlier identified in the parity plots of Figure 2a-b is the only phosphorus in the test set. The molecules containing the 5 phosphorus atoms in the training dataset are insufficient to predict the error of phosphorus subgraphs. To accurately extend this framework for accurately predicting the error of phosphorus subgraphs, a dataset of phosphorus-containing molecules should be used to refine the associated parameters of the model.

#
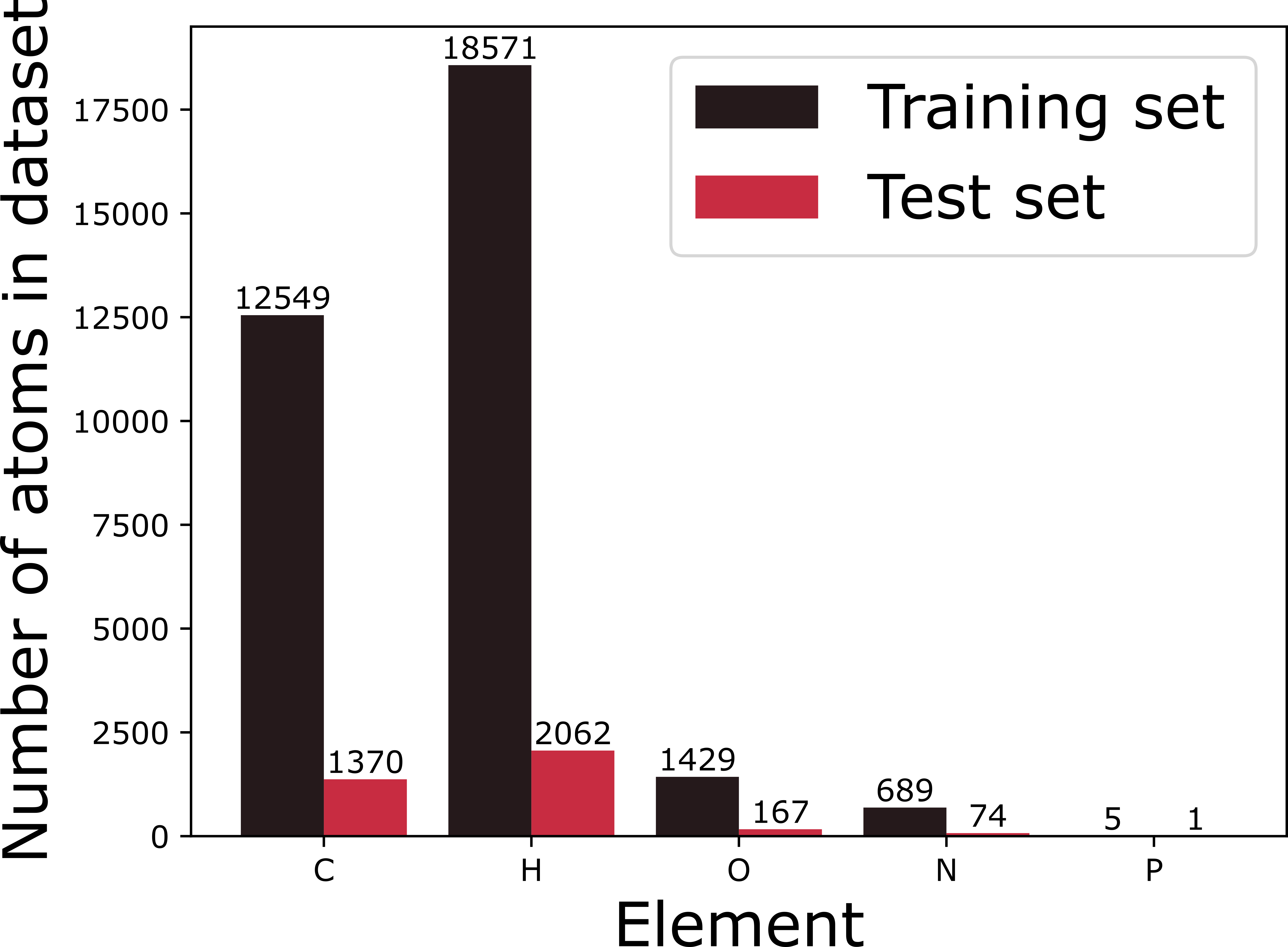


Figure S10. Number of element atoms in training and test sets.

# S6. Code and Data

Code and associated data of all the models are made available at: <https://github.com/VlachosGroup/Regularized-Machine-Learning-on-Molecular-Graph-Model-Explains-Systematic-Error-in-DFT-Enthalpies>
